# MRI-Guided Assessment of Rapid Locoregional Progression of Merkel Cell Carcinoma of the Thigh Following Wide Local Excision: A Case Report

**DOI:** 10.7759/cureus.116128

**Published:** 2026-09-12

**Authors:** Samia Obilat, Yahya Elharras, Hiba Zahi, Kaoutar Imrani, Ittimade Nassar

**Affiliations:** 1 Radiology Department, Ibn Sina University Hospital, Mohammed V University, Rabat, MAR

**Keywords:** locoregional progression, magnetic resonance imaging, merkel cell carcinoma, surgical margins, vascular encasement

## Abstract

Merkel cell carcinoma (MCC) is a rare and highly aggressive neuroendocrine skin cancer with a marked predilection for elderly and immunosuppressed patients. Although most commonly cutaneous, MCC can present with extensive locoregional soft tissue involvement, and its clinical course is characterized by rapid growth, frequent local recurrence, and early lymphatic and distant spread. We report the case of a 74-year-old woman who underwent wide local excision of a solitary thigh mass, with histopathology confirming MCC and a lateral margin that was uninvolved but immeasurably close. The patient was lost to follow-up shortly after surgery and, as a result, did not receive the recommended adjuvant treatment. She presented three months later with progressive swelling of the limb. Follow-up magnetic resonance imaging (MRI) revealed extensive, confluent cutaneous and subcutaneous masses spanning 32 cm along the thigh, infiltrating the adjacent muscular compartments, encasing the common femoral and common iliac vessels, and extending into the pelvis with intimate contact with the bladder wall and pelvic bones, without evidence of bone lysis. This dramatic progression, within only three months and in the absence of adjuvant therapy, rendered the lesion surgically unresectable. This case illustrates the aggressive natural history of MCC in an elderly patient lost to follow-up, highlights the central role of MRI in characterizing locoregional extension and supporting the assessment of surgical unresectability, and underscores the importance of adjuvant radiotherapy following excisions with close margins to prevent such outcomes.

## Introduction

Merkel cell carcinoma (MCC) is a rare, aggressive primary cutaneous neuroendocrine carcinoma, predominantly affecting elderly and/or immunosuppressed individuals, with a well-established association with the Merkel cell polyomavirus (MCPyV) and cumulative ultraviolet exposure [1,2]. Despite its rarity, MCC carries a disproportionately poor prognosis compared with other skin cancers, owing to its propensity for rapid local growth, frequent locoregional recurrence, and early lymphatic and hematogenous dissemination [3,4].

While the diagnosis of MCC relies on histopathological and immunohistochemical confirmation, cross-sectional imaging, particularly magnetic resonance imaging (MRI), plays an increasingly important role in delineating the extent of locoregional disease, especially in extremity involvement, where accurate assessment of muscular, vascular, and osseous involvement is essential for surgical planning and prognostication [5,6]. Imaging literature specifically describing the pattern and pace of locoregional progression of MCC in the soft tissues of the limb remains scarce [7,8].

We report a case of MCC of the thigh in which sequential MRI performed three months apart captured an unusually rapid locoregional progression, from a solitary, well-circumscribed lesion to extensive, multifocal, and ultimately unresectable disease, in a patient who was lost to follow-up after wide local excision and consequently did not receive adjuvant treatment. This longitudinal imaging comparison, together with detailed histopathological correlation, constitutes the principal contribution of this report.

## Case presentation

A 74-year-old woman initially presented with a solitary soft tissue mass of the thigh. Baseline MRI, including fat-suppressed T2-weighted and contrast-enhanced fat-suppressed T1-weighted sequences, revealed a well-circumscribed, lobulated subcutaneous mass located in the anteromedial aspect of the proximal thigh, with heterogeneous enhancement following gadolinium administration. The lesion measured approximately 75 x 120 mm in its anteroposterior and transverse diameters, extended over roughly 6 cm craniocaudally, and reached contact with the superficial femoral artery at its proximal margin, beyond which the vessel could no longer be clearly individualized. The mass was located at a distance from the femoral vein, along the expected course of the great saphenous vein, which could not be separately identified at this level (Figure 1).

**Figure 1:**
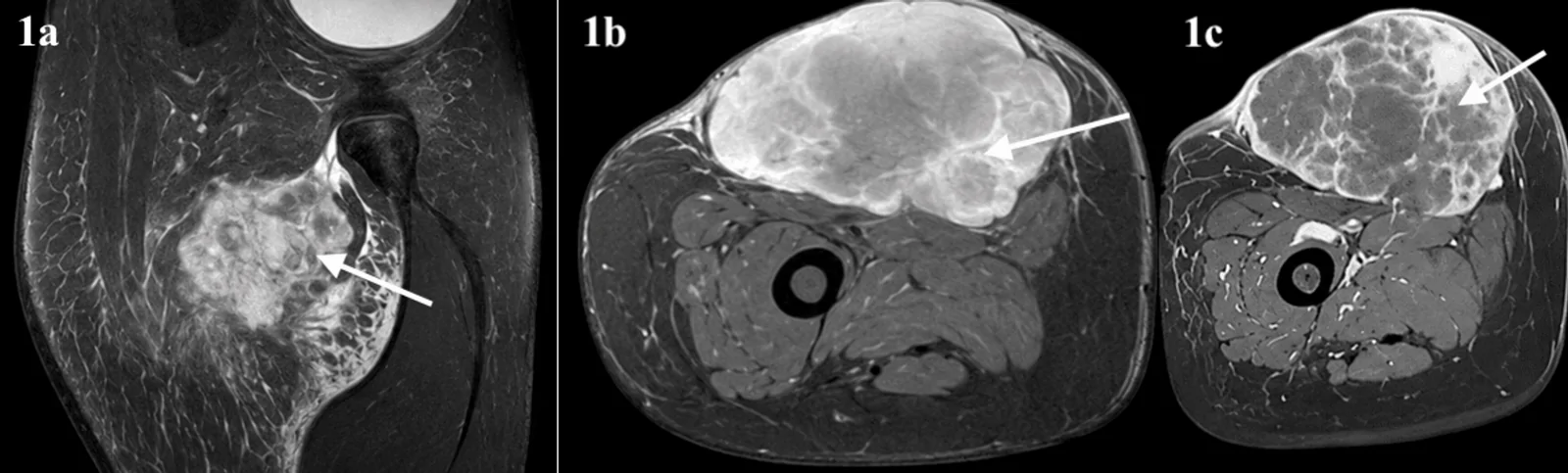
Baseline MRI of the solitary thigh mass Coronal (a) and axial (b) fat-suppressed T2-weighted images, and axial contrast-enhanced fat-suppressed T1-weighted image (c). White arrows indicate the solitary, lobulated subcutaneous mass in the proximal thigh, showing heterogeneous enhancement on the post-contrast image (c). The mass is separate from the superficial femoral vein and is located along the expected course of the great saphenous vein, which could not be clearly identified. MRI: magnetic resonance imaging

The patient underwent wide local excision. Pathological examination confirmed a 14 cm ulcerated, infiltrative tumor with an estimated 60% necrosis, infiltrating the dermis and subcutaneous tissue. Histology showed a solid and nodular proliferation of monomorphic, medium-sized round cells with scant basophilic cytoplasm and finely stippled, non-nucleolated chromatin. The nearest lateral margin was uninvolved but immeasurably close, while the other margins ranged from 1 to 3.5 cm. No vascular emboli or perineural invasion were identified (Figure 2).

**Figure 2:**
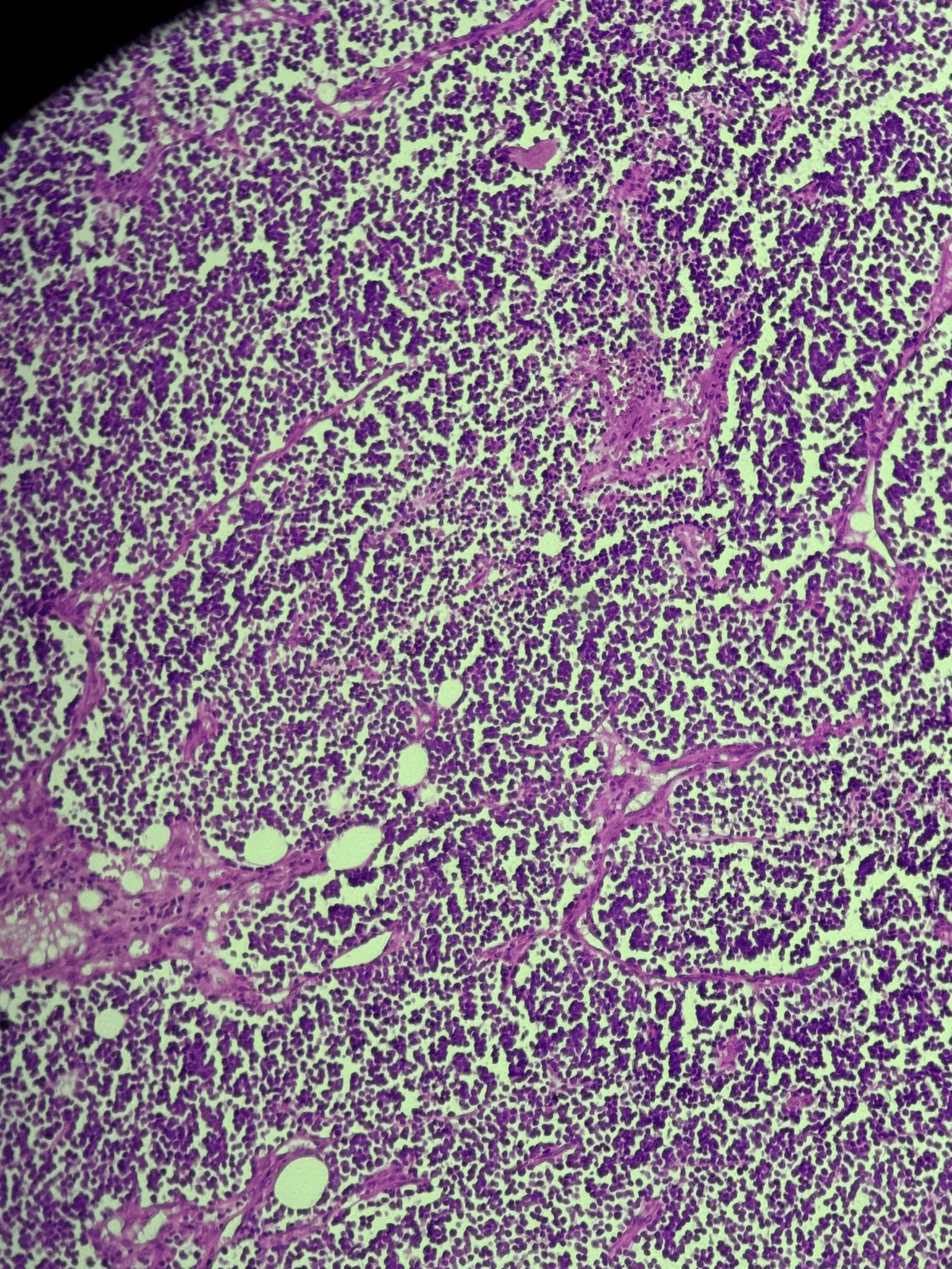
Histopathological features of Merkel cell carcinoma Hematoxylin and eosin staining showing a solid and nodular proliferation of monomorphic, medium-sized round cells with scant basophilic cytoplasm and finely stippled, non-nucleolated chromatin (original magnification x100).

Immunohistochemistry showed positive membranous and dot-like staining for AE1/AE3 and CK20, along with positivity for synaptophysin and chromogranin, confirming a neuroendocrine immunophenotype. CD20 and CD5 were negative, excluding a lymphoproliferative process, and PS100 was negative, excluding melanoma. This immunoprofile was consistent with MCC (Figure 3).

**Figure 3:**
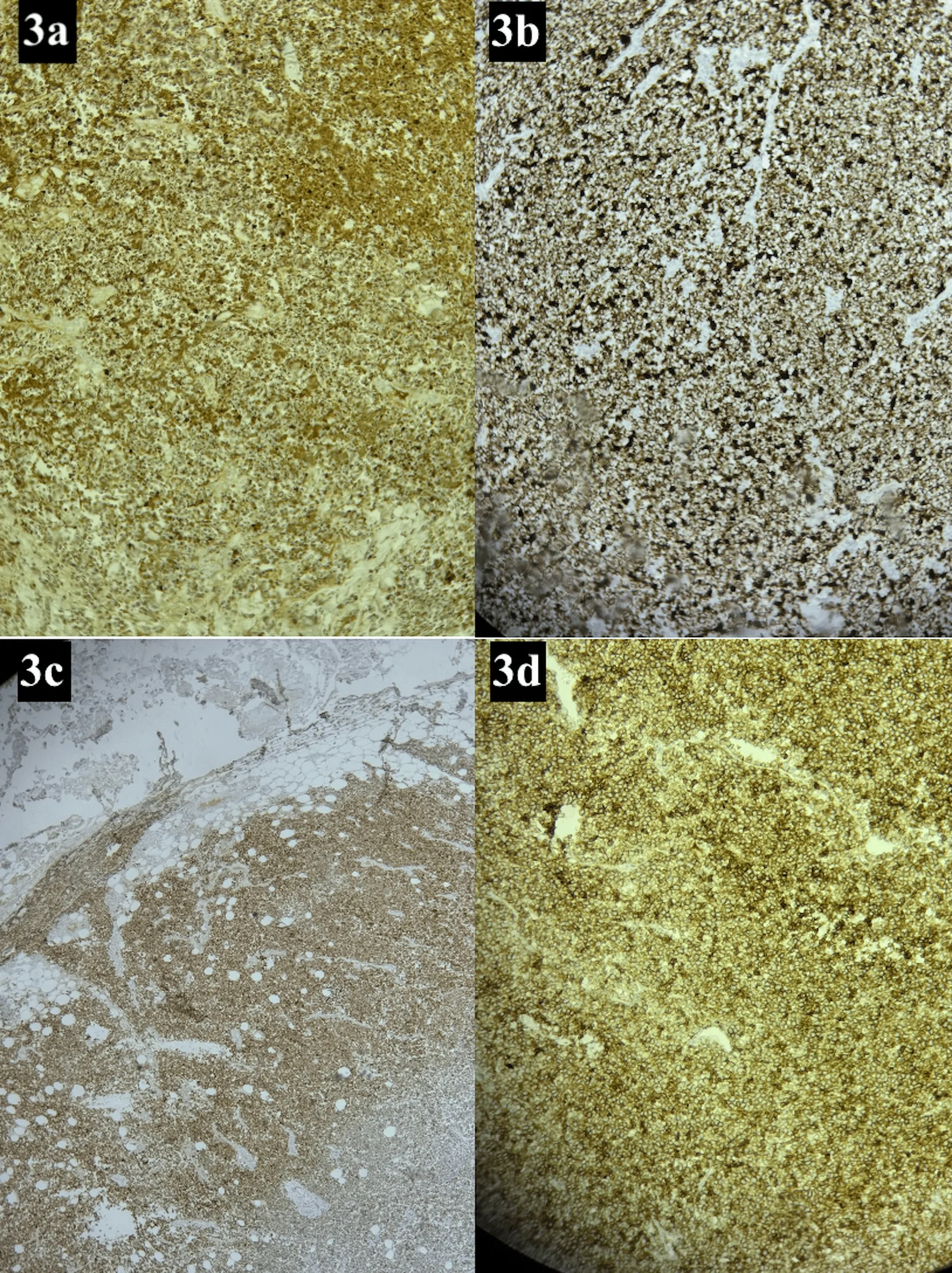
Immunohistochemical profile confirming Merkel cell carcinoma (a) AE1/AE3 immunostaining showing membranous and characteristic dot-like paranuclear positivity. (b) CK20 immunostaining demonstrating the characteristic dot-like paranuclear staining pattern. (c) Chromogranin immunostaining showing diffuse cytoplasmic positivity. (d) Synaptophysin immunostaining showing diffuse cytoplasmic positivity, further supporting the neuroendocrine phenotype.

No baseline nodal or systemic staging investigations, such as sentinel lymph node biopsy, contrast-enhanced CT, or PET-CT, were documented in the available clinical record. The patient was lost to follow-up shortly after surgery and therefore did not receive the recommended adjuvant treatment. She presented again three months later with progressive swelling of the entire lower limb.

Follow-up MRI revealed multiple confluent, lobulated cutaneous and subcutaneous masses extending along the anteromedial thigh to the inguinal region and labia majora, spanning approximately 32 cm. The lesions demonstrated heterogeneous T2/short tau inversion recovery (STIR) hyperintensity with areas of necrosis, T1 hypointensity, restricted diffusion, and heterogeneous post-contrast enhancement (Figure 4).

**Figure 4:**
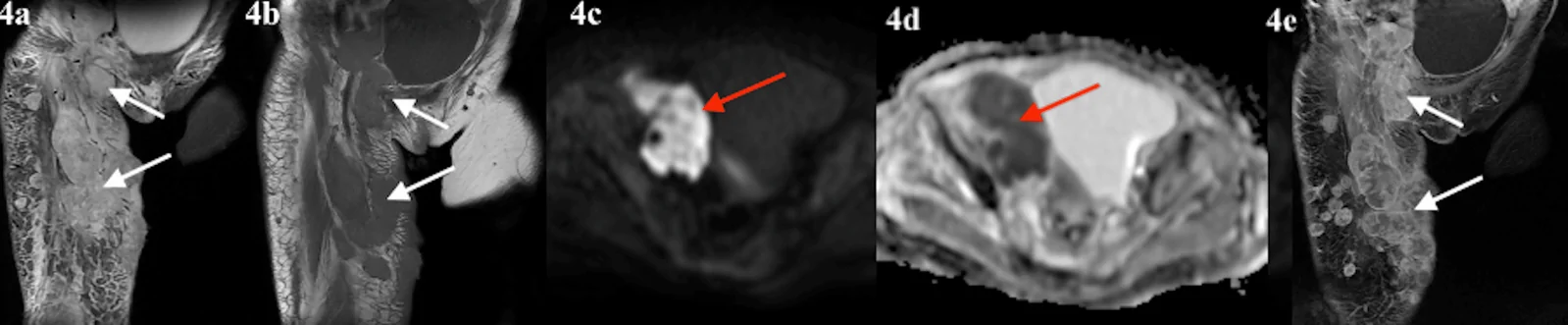
Follow-up MRI showing extensive locoregional progression from the pelvis to the thigh (a) Coronal STIR image showing confluent, lobulated hyperintense masses (white arrows) extending from the pelvis to the thigh, with extensive muscular and subcutaneous infiltration. (b) Corresponding coronal T1-weighted image (white arrows). (c) Axial diffusion-weighted image (b1000) at the pelvic level, showing the restricted diffusion component (red arrow) at the site of contact between the mass and the bladder, with encasement of the external iliac vessels. (d) Corresponding ADC map confirming true restricted diffusion within the same region (red arrow). (e) Coronal post-contrast image showing heterogeneous enhancement of the infiltrative masses (white arrows). STIR: short tau inversion recovery; MRI: magnetic resonance imaging; ADC: apparent diffusion coefficient

These masses extensively infiltrated the adjacent muscular compartments and subcutaneous soft tissues, with associated skin thickening. Pelvic extension was noted, with the masses encasing the common iliac, external iliac, and common femoral vessels, all of which remained patent, and reaching contact with the femoral vein, which also remained patent. The great saphenous vein showed thrombosis in places, while the deep femoral vein was not involved by tumoral extension. Intimate contact with the right lateral bladder wall without endoluminal extension, infiltration of the right transversus abdominis muscle, and contact with the iliac muscle and pelvic bones without osseous lysis were also noted (Figure 5).

**Figure 5:**
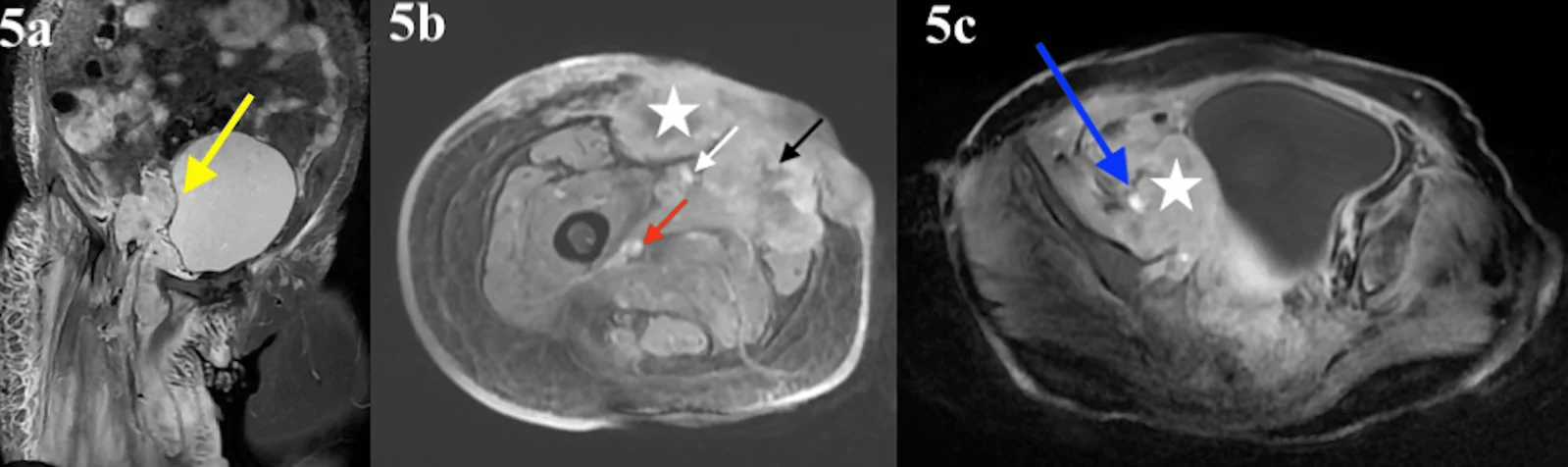
Pelvic extension with vascular involvement and bladder contact (a) Coronal image showing the pelvic component of the mass reaching intimate contact with the right lateral bladder wall (yellow arrow), without endoluminal extension. (b) Axial post-contrast image at the thigh level showing the mass (white star) reaching contact with the femoral vein (white arrow), which remained patent, with associated thrombosis of the great saphenous vein (black arrow); the deep femoral vein (red arrow) is not involved by tumoral extension. (c) Axial post-contrast image showing the mass (white star) encasing the external iliac vein (blue arrow), with preserved luminal patency.

Given the extent of vascular encasement, the lesion was considered surgically unresectable at this stage. No further histopathological sampling was performed; this progression was therefore inferred from clinical and radiological findings rather than confirmed by repeat histopathology, though it was judged highly consistent with locoregional progression of the previously confirmed MCC. The patient was subsequently referred for further oncologic management; however, details of the specific treatment administered and the long-term clinical outcome were not available in the records reviewed for this report.

## Discussion

MCC predominantly affects patients over 70 years of age, with advanced age itself constituting a recognized independent adverse prognostic factor, related in part to immunosenescence in a tumor known for its strong immunogenicity and responsiveness to immune-mediated control [9-11]. Beyond age, tumor viral status is increasingly recognized as an independent prognostic determinant: approximately 80% of MCC cases arise from clonal integration of the MCPyV, while the remainder are driven by cumulative ultraviolet-induced DNA damage. Virus-negative tumors have been shown to carry a significantly higher risk of disease progression and MCC-specific death compared with virus-positive tumors, independent of age, sex, and immunosuppression status [12]. Surgical margin status is another well-established determinant of outcome, with positive or close margins associated with higher rates of local recurrence and progression [3]. In our patient, two of these adverse factors, advanced age and an uninvolved but immeasurably close resection margin, were clearly present, offering a plausible explanation for the unusually fulminant course observed.

The differential diagnosis of small round blue cell tumors includes lymphoma, melanoma, small cell carcinoma, Ewing sarcoma/peripheral primitive neuroectodermal tumor, and other neuroendocrine neoplasms [13,14]. Immunohistochemistry is therefore essential for diagnosis. In our case, tumor cells showed the characteristic dot-like paranuclear positivity for AE1/AE3 and CK20, together with synaptophysin and chromogranin positivity, confirming neuroendocrine differentiation. Negative CD20, CD5, and PS100 staining helped exclude a lymphoproliferative process and melanoma [15,16]. Combined with the clinical and imaging findings, this immunophenotype was considered diagnostic of MCC.

MRI is the modality of choice for assessing the soft tissue extent of MCC in the extremities, owing to its superior contrast resolution for differentiating tumor from adjacent muscle, fat, and neurovascular structures [5,6,17]. MCC typically appears as a well-defined mass that is iso- to hypointense to skeletal muscle on T1-weighted images, hyperintense on T2/STIR sequences, and shows homogeneous avid enhancement at early stages, with increasing heterogeneity, central necrosis, and restricted diffusion as the tumor burden and grade of aggressiveness increase [18]. This progression was well illustrated in our patient: the baseline lesion was relatively well circumscribed but already demonstrated heterogeneous enhancement, whereas the follow-up study showed a confluent, necrotic, and markedly heterogeneous process. Sequential MRI in this case therefore provided an unusually clear illustration of the pace and pattern of disease progression, from a solitary, well-circumscribed mass to a diffuse, multifocal, and confluent process with muscular infiltration, vascular encasement, and pelvic extension, all within three months. Beyond simple lesion characterization, MRI provided critical anatomical information in this patient's management: by precisely delineating the relationship of the tumor to the femoral and iliac vessels, the bladder wall, and the pelvic musculature, it supported the assessment of surgical unresectability and informed the reorientation of the therapeutic strategy toward radiotherapy and systemic treatment. This case therefore illustrates how detailed MRI staging can meaningfully inform oncologic management in MCC, extending its role well beyond initial diagnosis to encompass surgical planning, monitoring for progression, and treatment decision-making, underscoring the central place of cross-sectional imaging, and MRI in particular, in the multidisciplinary management of this aggressive tumor.

Current guidelines recommend wide local excision with sentinel lymph node biopsy for clinically node-negative MCC, followed by adjuvant radiotherapy to the primary site and regional nodal basin, particularly in the setting of close or positive margins, given the tumor's propensity for local recurrence even after apparently complete resection [4,19]. In this patient, loss to follow-up shortly after surgery precluded the administration of adjuvant treatment altogether, which may have contributed, alongside other adverse prognostic factors, to the rapid and extensive locoregional progression observed; however, a single case cannot establish that this omission was causal or that adjuvant radiotherapy would necessarily have prevented this outcome. Once vascular encasement renders the disease surgically unresectable, treatment options shift toward definitive or palliative radiotherapy and systemic therapy, including immune checkpoint inhibitors targeting the PD-1/PD-L1 axis, such as avelumab and pembrolizumab, which have transformed outcomes in advanced or metastatic MCC [4,9]. In the pivotal JAVELIN Merkel 200 trial, avelumab produced durable objective responses in approximately one-third of patients with chemotherapy-refractory metastatic MCC, with the majority of responses maintained beyond three years, representing a marked improvement over historical outcomes with cytotoxic chemotherapy, which rarely provided disease control beyond a few months [20]. This case reinforces the importance of close adherence to adjuvant treatment recommendations and structured follow-up in patients with adverse margin status.

## Conclusions

This report illustrates the potentially explosive natural history of MCC, progressing from a solitary thigh mass to extensive, unresectable locoregional disease within three months in an elderly patient with a close resection margin, lost to follow-up, and without adjuvant treatment. Although causality cannot be established from a single observation, this course occurred alongside several recognized adverse prognostic factors, including advanced age and a close margin. MRI proved essential in characterizing the extent of locoregional spread and supporting the assessment of surgical unresectability, underscoring the importance of guideline-concordant adjuvant treatment and close surveillance in MCC with adverse margin status.
